# Association of Metals and Metalloids With Urinary Albumin/Creatinine Ratio: Evidence From a Cross-Sectional Study Among Elderly in Beijing

**DOI:** 10.3389/fpubh.2022.832079

**Published:** 2022-03-31

**Authors:** Ang Li, Jiaxin Zhao, Liu Liu, Yayuan Mei, Quan Zhou, Meiduo Zhao, Jing Xu, Xiaoyu Ge, Qun Xu

**Affiliations:** ^1^Department of Epidemiology and Biostatistics, Institute of Basic Medical Sciences Chinese Academy of Medical Sciences, School of Basic Medicine Peking Union Medical College, Beijing, China; ^2^Center of Environmental and Health Sciences, Chinese Academy of Medical Sciences, Peking Union Medical College, Beijing, China; ^3^Chaoyang District Center for Disease Control and Prevention, Beijing, China

**Keywords:** urinary Cu, kidney function, UACR, elderly, quantile g-computation, BKMR

## Abstract

**Background:**

Environmental exposure to toxic elements contributes to the pathogenesis of chronic kidney disease (CKD). Few studies focus on the association of urinary metals and metalloids concentrations with the urinary albumin/creatinine ratio (UACR) among elderly, especially in areas and seasons with severe air pollution.

**Objective:**

We aimed to evaluate the associations of urinary metals and metalloids concentration with UACR, which is an early and sensitive indicator of CKD.

**Method:**

We conducted a cross-sectional study among 275 elderly people in Beijing from November to December 2016, which has experienced the most severe air pollution in China. We measured 15 urinary metals and metalloids concentration and estimated their association with UACR using a generalized linear model (GLM). Bayesian kernel machine regression (BKMR) and quantile g-computation (qgcomp) models were also conducted to evaluate the combined effect of metal and metalloid mixtures concentration.

**Results:**

Of the 275 elderly people included in the analysis, we found that higher urinary Cu concentration was positively associated with UACR using GLM (β = 0.36, 95% CI: 0.25, 0.46). Using the BKMR model, we found that the change in UACR was positively associated with a change in urinary Cu concentration from its 25th to 75th percentile value with all other metals and metalloids concentration fixed at their 25th, 50th, or 75th percentile levels. Urinary Cu concentration had the most significant positive contribution (59.15%) in the qgcomp model. Our finding was largely robust in three mixture modeling approaches: GLM, qgcomp, and BKMR.

**Conclusion:**

This finding suggests that urinary Cu concentration was strongly positively associated with UACR. Further analyses in cohort studies are required to corroborate this finding.

## Introduction

Chronic kidney disease (CKD) is a global public health problem that imposes heavy burden on both developed and developing countries (1). The overall prevalence of CKD in China increased to 10.8% (2), and global CKD-related mortality rates increased by ~14% from 1990 to 2010 (9.6-1.1 per 100,000) (3). As an organ with the ability to metabolize, the kidney is a target organ for metal and metalloid toxicity (4), which can accumulate in the nephron, leading to renal dysfunction. The 2012 Kidney Disease Improving Global Outcome (KDIGO) guidelines define CKD by estimated glomerular filtration rate (eGFR) or markers of kidney damage, such as albuminuria [defined as urinary albumin/creatinine ratio (UACR) ≥ 30 mg/g)] (5). Clinically, UACR has been considered an early and sensitive indicator to determine CKD (6). UACR is beneficial in that it has low requirements for samples, as it is noninvasive and can be detected using a spot urine sample rather than 24 h urine (7).

Metals and metalloids may be contained in soil, drinking water, food, ambient air and consumer products (8–14). For example, Pb released into the environment by petrol, domestic Pb-based paints and cigarette smoke (15, 16). Exposure to Cu and Se is mainly dietary, and both are naturally found in seafood, nuts, rice, whole grains, and some fruits and vegetables (17). The general population can also be exposed to Mn *via* traffic-related emissions, due to the use of Mn as an antiknock additive in gasoline (18). It is difficult to evaluate the real exposure of human body through a single or several external exposure media (i.e., air or water). Previous studies have reported that metals can enter the body through oral, inhalational, or transdermal routes, they circulate in the blood and are excreted in the kidney as urine (19–21). Urinary metal concentrations were mostly regarded as a reliable indicator of exposure as they can integrate multiple exposure sources (22, 23). Therefore, the study assessed human exposure to multiple metals by detecting metals in urine.

Metals and metalloids are known to be a risk factor of CKD (24), which may result in end-stage renal disease and increase the risk for all-cause mortality and cardiovascular disease (25). Although several studies have suggested an association between metals and metalloids concentration and kidney function (26–28), studies focusing on the possible relationship between multiple metals and metalloids concentration and UACR have been limited, especially among elderly. Additionally, humans are typically exposed to complex mixtures rather than single agents (29); thus, it is important to explore the health effects of metal and metalloid mixtures.

The traditional generalized linear model (GLM) was broadly used to estimate the association of a single metal or metalloid concentration with health outcomes based on the hypothesis that the association was linear. In recent years, weighted quantile sum (WQS) regression, quantile g-computation (qgcomp) and Bayesian kernel machine regression (BKMR) model have been proposed to investigate the effects of combined exposure. To further assess the contributing effects of individual metals or metalloids, WQS regression analysis was employed to estimate the combined and discrete effects of multiple predictors in the context of correlated high-dimensional mixtures (30). However, WQS regression should be based on the assumption of directional homogeneity, and individual exposures have linear and additive effects as well (31). A study reported that qgcomp is an adaptive method of WQS regression to estimate the association of metals and metalloids in mixtures in environmental epidemiology (32). The method has advantages over WQS, including that directional homogeneity of effect estimates is unnecessary (33). Considering the possibility of nonlinearity and interaction, BKMR was proposed to explore the relationship between metal and metalloid mixtures and health outcomes. Although these methods are gradually being applied, few studies have compared these models together to understand the stability of the results.

In this cross-sectional study, we selected 275 elderly who lived in Beijing for at least 1 year by November and December 2016, the season with the most severe air pollution. The association of multiple urinary metals and metalloids and their mixtures with UACR was examined. We selected 15 metals and metalloids (aluminum [Al], arsenic [As], barium [Ba], cadmium [Cd], cobalt [Co], chromium [Cr], cesium [Cs], copper [Cu], iron [Fe], manganese [Mn], nickel [Ni], lead [Pb], selenium [Se], strontium [Sr] and zinc [Zn]) based on demonstrated developmental nephrotoxicity in animal models (34, 35) and evidence from existing literature that identified associations with kidney parameters in people (19, 36, 37). We applied multiple models, including the GLM, BKMR and qgcomp models, to estimate the association of urinary metals and metalloids concentration with UACR and to explore the stability of the results among different models.

## Materials and Methods

### Study Design and Participants

We conducted a cross-sectional study and utilized data based on communities distributed from south to north in Beijing, which experienced the nation's highest levels of PM_2.5_ during the last 20 years (38). In 2016, the annual average concentration of PM_2.5_ reached 73 μg/m^3^ in Beijing according to the Ministry of Environmental Protection of China, while the China National Air Quality Standard for PM_2.5_ is 35 μg/m^3^ (39). Medical examinations and testing were administered to participants between November and December 2016 during the winter heating period, when Beijing became a hot spot for anthropogenic heavy metal emissions with the increase in coal burning, worsening the air pollution situation (40). Eligible participants were above 60 years of age and lived at least 1 year. We excluded subjects who were unable to complete anthropomorphic examinations or questionnaires. Furthermore, we excluded participants who had malignant tumors, liver disease and endocrine disease. We further excluded participants with missing or insufficient urine samples for detection, as well as missing other variables of interest. A total of 275 subjects were finally included in the analyses. Written informed consent was obtained from all participants. In addition, the study was approved by the institutional ethics committees of the Institute of Basic Medicine in the Chinese Academy of Medical Sciences.

### Determination of Urinary Metal and Metalloid Concentrations and UACR

Morning first void urine samples were collected in polypropylene containers and stored at −80°C. We measured 15 urinary metals and metalloids, including Al, As, Ba, Cd, Co, Cr, Cs, Cu, Fe, Mn, Ni, Pb, Se, Sr and Zn, using inductively coupled plasma-mass spectrometry (ICP-MS) with a Nexion 300D (Perkin-Elmer SCIEX, USA), which has been described in detail in previous study (41). The urine samples were thawed at room temperature for subsequent processing. One milliliter of urine was made up to 15 mL with 0.5% (v/v) HNO_3_ and 0.02% Triton X-100 and treated by sonication in an ultrasonic water bath for 1 h at 60°C. In terms of quality control procedures, urinary metals and metalloids were measured three times and averaged for analysis. Rhodium (Rh) element standard solution (Central Iron and Steel Research Institute, National Testing Center for Iron and Steel Materials) was used as the internal standard solution, diluted to 10.0μg/L with blank solution. Besides, the urine quality control samples of trace element (Trace Element Urine L-1 ROU, REF: 210613, LOT: 1706877, Seronorm, Norway) was used as certificated reference material (CRM), and the measured concentrations of each metal is within the 95 % CI provided by CRM. Moreover, the spiked recovery samples were measured every 20 specimens to ensure the correct analysis of urine samples. Only when the quality control sample is within the normal range (e.g., the recovery rate is in the range of 80-120%) will we proceed with the subsequent analysis. Additionally, the intra-assay and inter-assay coefficients were <10% (see Supplementary Tables S1, S2 for more validation parameters for urinary metals and metalloids). The value below the limit of detection (LOD) was assigned LOD/2.

Considering the concentration dilution of urine, the concentrations of urinary metals and metalloids were all corrected by urinary creatinine. Creatinine-adjusted urinary metal and metalloid concentrations were included in subsequent analyses. We measured urinary creatinine and albumin (mg/dL) on a Beckman Coulter analyzer (AU5800 Analyzer, Beckman Coulter, Brea, CA, USA) from morning first void spot samples. Urinary creatinine was measured by the sarcosine oxidase method, and albumin was measured by immunoturbidimetry. UACR (mg/g), which is an indicator of albuminuria, was determined. Elevated UACR is defined as an abnormally high urine albumin concentration (42).

### Covariate Assessment

Information on demographic characteristics and lifestyle factors was collected by registered physicians using standard questionnaires, including age, sex (male/female), education level (primary school or below, middle school, high school or above), smoking status (never, current, former), drinking status (never, current, former), hypertension (yes/no) and diabetes (yes/no). Current smokers were identified as smoking more than one cigarette or cigar or water pipe/day over the last 6 months. Current drinking was identified as drinking alcoholic beverages at least once per week over the last 6 months. Former smokers/drinkers were identified as quitting smoking/drinking for more than six months.

Participants were examined according to a standardized protocol by registered physicians, including weight, height, systolic blood pressure (SBP) and diastolic blood pressure (DBP). BMI (kg/m^2^) was calculated as weight divided by height squared. Blood pressure was measured on the right arm after a 15 min rest period in the sitting position by a mercury sphygmomanometer. SBP and DBP were calculated as follows: if the second and third measurements are <5 mmHg, take the average value; otherwise, re-measure until the difference is <5 mmHg. Hypertension was defined using SBP≥140 mmHg and/or DBP≥90 mmHg, intake of any antihypertensive drug, or both (43). Diabetes was defined using fasting glucose ≥7.0 mmol/L, 2 h glucose ≥11.1 mmol/L, intake of any antidiabetic drug, or both (44). According to the KDIGO (45), (a) an eGFR of <60 mL/min/1.73 m^2^, (b) albuminuria (defined as UACR ≥ 30 mg/g), or (c) a medication regimen for treating renal dysfunction is sufficient for CKD diagnosis (46).

### Statistical Analysis

Descriptive statistics were used to describe the frequency and proportion of the demographic and clinical characteristics. We described normally distributed data using means with standard deviations (SD), and the median and interquartile range (IQR) were used to describe data with skewness distribution. Both creatinine-adjusted urinary metals and metalloids and UACR were required to logarithmically convert to an approximate normal distribution.

#### GLM Analysis

GLMs were used to analyze the association of individual urinary metal and metalloid concentrations with UACR. We conducted linear regression to explore the associations [estimated by regression coefficient β and its 95% confidence interval (CI)] between log–transformed urinary metal and metalloid concentrations as continuous independent variables and UACR as continuous dependent variables. Multiple comparisons were accounted for using the Benjamini and Hochberg method for false discovery rates (FDR) (47). In addition, the urinary metal concentrations were divided into 3 categories in terms of the tertile distribution. The tertile with the lowest value of urinary metal concentration was regarded as reference, regression coefficient β and 95% CI were also reported to indicate the association between urinary metal concentration and UACR in each tertile, respectively. Based on prior knowledge and literature accumulation (7, 48, 49), we adjusted age, sex, BMI, education levels, smoking status, drinking status, hypertension and diabetes in the model.

#### BKMR Analysis

To further consider the possibility of nonlinearity and interaction and explore the health effects of metals and metalloids in mixtures, we also conducted a BKMR model to estimate the associations between metal concentration and kidney function (50). Combining Bayesian and statistical learning methods, BKMR was introduced to regress an outcome variable on a nonparametric term of exposure mixture (50). We explored the overall association of metal and metalloid mixtures concentration in relation to UACR and conducted the dose-response association of single metals and metalloids, as other metals and metalloids concentration were fixed at the 50th percentile. In addition, we estimated the risk difference between a single exposure at the 75th percentile and an exposure at the 25th percentile, and all remaining metals were fixed at the 25, 50, or 75th percentile level. We also further explored the specific interactions exposed on UACR. In addition, we estimated risk differences when a single metal or metalloid was at the 75th percentile compared to 25th percentile, and other metals and metalloids were fixed at their 25, 50, or 75th percentile levels. The specific interactions between metals and metalloids on UACR were further explored. We used Markov chain Monte Carlo algorithm to implement BKMR variable selection model with 25,000 iterations. The confounding factors that were adjusted in the BKMR were the same as those in the GLM.

#### qgcomp Analysis

Individual associations of metals and metalloids in the mixture with health outcome can be inverse; thus, qgcomp was introduced. Unbiased estimation of the overall mixture effects is produced within the coverage of small sample size and acceptable CI. qgcomp used a parametric GLM-based application of g-calculation to evaluate the effect of increasing all metals and metalloids in the mixture by a quarter simultaneously (31). The advantage of qgcomp is that metals and metalloids can interact with outcomes in any direction (21). This method is applied in following steps. First, all elements were transformed into quantiles. Second, a linear model was fit between the elements, UACR and covariates, including age, sex, BMI, education levels, smoking status, drinking status, hypertension and diabetes. Third, weights are defined for each element, corresponding to the strength of the relationship between the element and UACR. The overall effect of the mixture can be interpreted as the change in UACR per quantile of change in all elements while controlling for covariates. If the element has different effects in different directions, the positive or negative weights are interpreted as the percentage of exposure effects that have a negative (or positive) effect on UACR.

#### Sensitivity Analyses and Stratify Analysis

Considering that CKD status may be a collider between urinary metals and UACR, we did not adjust for CKD status in the main model. However, to examine whether CKD status may confound the association between urinary elements and UACR, we further added the CKD status (yes/no) in sensitivity analyses. We also eliminated the outliers of urinary Cu which above the mean concentration ±3 SD to explore the stability of the results.

In the stratified analysis, we conducted the stratified analysis by age (<65 years/above age 65 or more), gender (male/female), smoking status (yes/no), and drinking status (yes/no). Multiple comparisons were also accounted by P_FDR_.

All analyses were carried out in R software (R version 3.6.2) and with the packages “bkmr”, “gqcomp” and “ggplot” for plotting the quantification and visualization results of the BKMR model.

## Results

### Demographic Characteristics

Table 1 presents the demographic and clinical characteristics of the 275 study participants (122 men and 153 women), with a mean age of 68.9 years and a mean BMI of 24.5 kg/m^2^. More than half of the participants never smoked (68.0%) or drank (57.1%). The median (25, 75th percentile) of clinical values were 6 (3, 18) mg/g UACR and 9.3 (5.5, 12.1) μg/g urinary creatinine. The LOD, geometric mean concertation (GM), geometric standard deviation (GSD) and distribution of 15 urinary elements are shown in Table 2. The concentrations of most urinary metals and metalloids were higher than the LOD.

**Table 1:** Baseline information of the study participants (*n* = 275).

**Characteristic**	**All participants (*n =* 275)**
Age [years], mean ± SD	68.9 ± 6.8
BMI [kg/m^2^], mean ± SD	24.5 ± 3.8
**Sex**, ***n*** **(%)**	
Male	122 (44.4)
Female	153 (55.6)
**Education**, ***n*** **(%)**	
Primary school or below	131 (47.6)
Middle school	55 (20.0)
High school or above	87 (31.6)
Refuse to answer	2 (0.7)
**Smoking status**, ***n*** **(%)**	
Never	187 (68.0)
Current	42 (15.3)
Former	41 (14.9)
Refuse to answer	5 (1.8)
**Drinking status**, ***n*** **(%)**	
Never	157 (57.1)
Current	21 (7.6)
Former	90 (32.7)
Refuse to answer	7 (2.5)
Urinary creatinine [μg/g], median (IQR)	9.3 (5.5–12.1)
UACR [mg/g], median (IQR)	6 (3–18)

*BMI, body mass index; IQR, interquartile range; SD, standard deviation; UACR, urinary albumin/creatinine ratio*.

**Table 2:** Detection rates and distribution for 15 adjusted urinary metals and metalloids.

**Element** **(μg/g creatinine)**	**%>LOD^**a**^**	**GM(GSD)^**b**^**	**Quartiles of adjusted urinary elements**			
			**25th**	**50th**	**75th**	**90th**
Al	100.00	4.358 (2.144)	2.484	4.264	7.274	12.776
As	99.99	2.063 (2.200)	1.377	1.954	3.347	5.235
Ba	99.97	0.307 (3.424)	0.169	0.288	0.776	1.445
Cd	99.95	0.049 (3.295)	0.038	0.066	0.109	0.153
Co	99.99	0.104 (2.386)	0.051	0.105	0.212	0.338
Cr	100.00	0.274 (1.668)	0.189	0.267	0.381	0.569
Cs	100.00	0.947 (1.898)	0.716	0.966	1.324	1.724
Cu	99.99	1.082 (1.750)	0.749	1.093	1.601	2.118
Fe	100.00	3.903 (2.096)	2.170	3.850	6.474	10.753
Mn	61.80	0.031 (4.236)	0.008	0.027	0.094	0.235
Ni	100.00	0.311 (3.202)	0.188	0.375	0.736	1.077
Pb	99.86	0.118 (3.831)	0.042	0.141	0.325	0.631
Se	100.00	3.391 (2.153)	1.667	3.926	6.168	8.911
Sr	100.00	11.499 (1.909)	7.926	11.872	18.078	24.720
Zn	100.00	46.847 (1.776)	32.610	47.838	68.342	102.643

a *% > LOD, detection rate above limit of detection*.

b *GM, geometric mean; GSD, geometric standard deviation*.

### Results of Main Analysis

#### Association Between Urinary Element Concentrations and UACR in GLM

The relationships between individual adjusted urinary elements and UACR in GLM are shown in Table 3. We observed that urinary Cu, Fe and Zn concentrations were positively associated with UACR after adjustment for age, sex, BMI, education levels, smoking status, drinking status, hypertension and diabetes in a single metal and metalloid model. Urinary Cu had the highest estimate effect (β = 0.36, 95% CI: 0.25 to 0.46; *R*^2^ = 0.39, *P-*value = 2.82 × 10^−10^), followed by Fe (β = 0.12, 95% CI: 0.02 to 0.22; *R*^2^ = 0.27, *P-*value = 0.02) and Zn (β = 0.14, 95% CI: 0.01 to 0.28; *R*^2^ = 0.27, *P-*value = 0.04). After FDR adjustment, only urinary Cu concentration was remained significantly and positive associated with UACR (*P*_FDR_ < 0.05). Comparing with the lowest tertile, the total population demonstrated significant increased UACR in the third tertile of urinary Cu (β = 0.55, 95% CI: 0.37-0.73) and urinary Fe (β = 0.29, 95% CI: 0.10-0.49) (Supplementary Table S3).

**Table 3:** Estimated associations between urinary elements and UACR in GLM^a^.

**Elements (μg/g creatinine)**	**β (95% CI)**	***R*** ^**2**^	***P-*** **value**	***P***_**FDR**_
Al	0.00 (−0.09, 0.09)	0.25	1.00	1.00
As	0.05 (−0.06, 0.16)	0.25	0.36	0.61
Ba	0.04 (−0.03, 0.10)	0.25	0.26	0.61
Cd	0.03 (−0.04, 0.10)	0.25	0.46	0.61
Co	0.03 (−0.06, 0.12)	0.25	0.53	0.61
Cr	0.03 (−0.10, 0.17)	0.25	0.64	0.68
Cs	0.09 (−0.04, 0.22)	0.26	0.18	0.61
Cu	**0.36 (0.25, 0.46)**	0.39	**<0.01**	**<0.01**
Fe	0.12 (0.02, 0.22)	0.27	0.02	0.14
Mn	−0.02 (−0.07, 0.03)	0.25	0.45	0.61
Ni	0.03 (−0.04, 0.11)	0.25	0.39	0.61
Pb	0.03 (−0.03, 0.09)	0.25	0.33	0.61
Se	0.06 (−0.05, 0.17)	0.25	0.29	0.61
Sr	0.04 (−0.08, 0.16)	0.25	0.53	0.61
Zn	0.14 (0.01, 0.28)	0.27	0.04	0.19

a *The Model was adjusted for age, sex, BMI, education levels, smoking status, drinking status, hypertension and diabetes*.

*Boldface type indicates effect estimates were statistically significant (p-value < 0.05)*.

#### Association Between Urinary Element Concentrations and UACR in qgcomp

**Figure 1:**
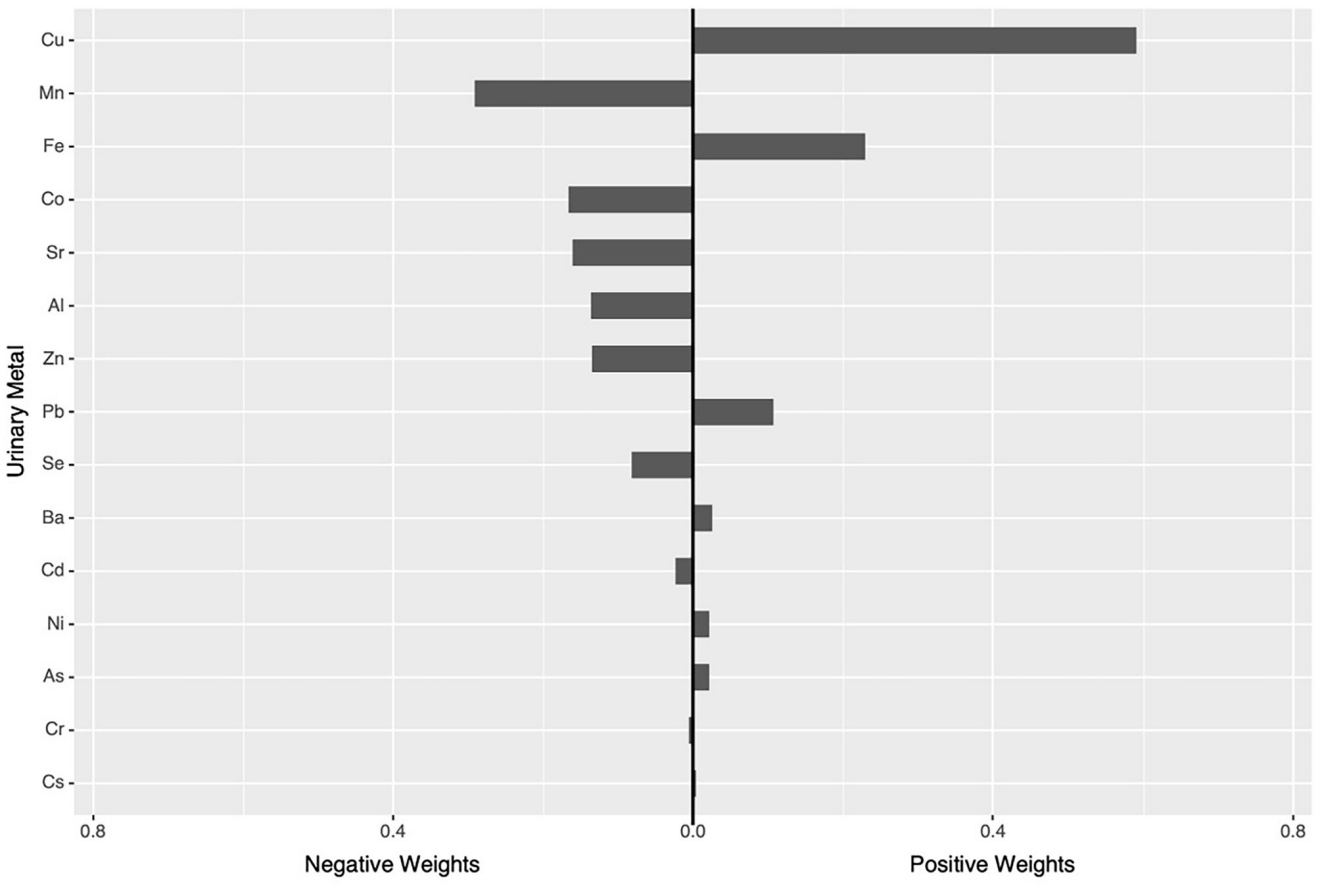
qgcomp model^a^ regression index weights for UACR. ^a^The Model was adjusted for age, sex, BMI, education levels, smoking status, drinking status, hypertension and diabetes.

In the qgcomp mixtures approach (Figure 1), a marginal association was observed between the urinary element mixture concentration and higher UACR (β = 0.07, 95% CI: −0.08, 0.22). Urinary Cu (59.15%) concentration had the greatest positive contribution to the overall effect, and urinary Mn (29.15%) concentration had the largest negative weight.

#### Association Between Urinary Element Concentrations and UACR in the BKMR Model

**Figure 2:**
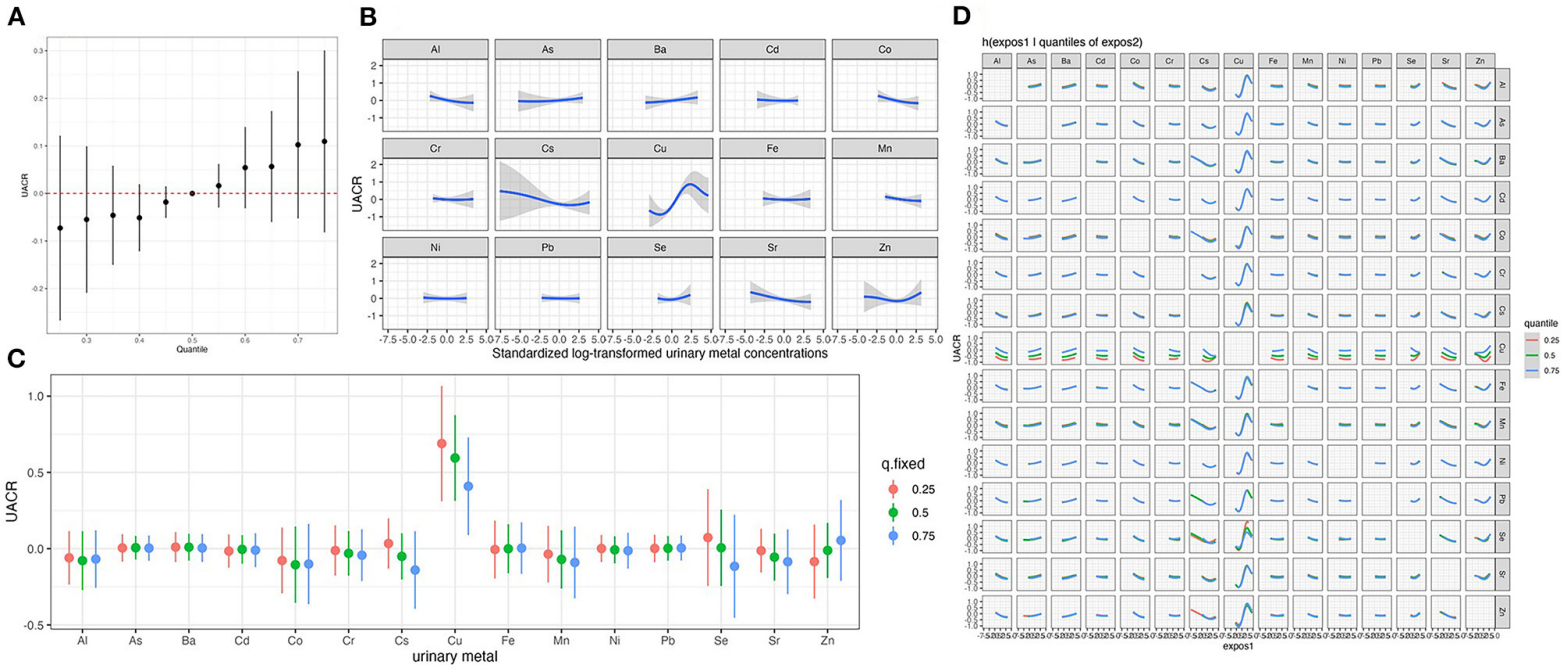
Joint effect of the urinary metal and metalloid mixture on UACR by Bayesian Kernel Machine Regression^a^. **(A)** Overall associations between the urinary metal and metalloid mixtures and UACR. **(B)** Univariate exposure-response curves for individual metal. **(C)** The single-exposure effect of each metal. **(D)** Bivariate exposure response functions for each of the urinary metal and metalloid. ^a^The Model was adjusted for age, sex, BMI, education levels, smoking status, drinking status, hypertension and diabetes.

We estimated the overall association analysis that regarded the urinary elements as a mixture and found a marginal positive correlation trend with UACR, which was similar to the result of qgcomp (Figure 2A). To further explore potential nonlinear relationships, we conducted univariate exposure-response functions (Figure 2B). The plot indicates a nonlinear association between urinary Cu concentration and UACR. We further estimated univariate summaries of the change in the UACR associated with a change in a single metal or metalloid from its 25th percentile to 75th percentile, where all of the other pollutants are fixed at a particular threshold (25, 50, or 75th percentile) (Figure 2C). Urinary Cu is the only element displaying a significant effect, and its positive association with UACR appears stronger at lower percentiles of other elements. An approximately S-shaped dose-response curve for Cu was observed, and the CI became wider as observations became less frequent for high and low Cu concentrations. The single-pollutant estimates from Figure 2D suggested that most elements have no interaction with each other except for Cu and Cs and Cu and Zn.

### Results of Sensitivity and Stratification Analysis

The results of the sensitivity analysis suggested that our findings were robust. Supplementary Figure S1 shows that urinary Cu remained significantly positively associated with UACR after additionally adjusting for CKD status (β = 0.17, 95% CI: 0.09, 0.25). We observed that the CI of the two tails in is very wide in Figure 2B, which may be due to the influence of outliers on the results. Thus, we excluded the outliers of urinary Cu concentration which above the mean concentration ±3 SD and found a linear positive association between urinary Cu and UACR in BKMR (Supplementary Figure S2).

Stratified analysis of our data showed significantly positive associations between urinary Cu and UACR in all stratified analyses (*P* < 0.05) (Supplementary Tables S4,S5). The estimated effects were higher in the above 65-year-old, female, smoking and drinking populations.

## Discussion

In this study, the association of multiple urinary elements and element mixtures concentration with UACR was investigated in typical severe air polluted areas and seasons. We found that urinary Cu concentration was significantly and positively associated with UACR in GLM, BKMR and qgcomp, which indicates that Cu was associated with an increased risk of albuminuria. To better understand the results, we also supplemented the assumptions, advantages and disadvantages of each model in supplementary (Supplementary Table S6).

To compare the concentration of urinary metals with published studies, we have searched and included several studies if they had an observational design; were conducted on general participants without environmental or occupational exposure; and reported data on exposure to metals of interest in this study (36, 51–54). By comparing with other studies (Supplementary Table S7), we found that most of urinary metals concentration in this study are lower than published studies, such as Cu, Al, Co, Fe, Mn and Sr. On the contrary, the concentrations of Cs, Se and Zn were higher than published studies. Besides, some metals, such As, Ba, Cd, Cr, Ni, and Pb, are in the range of published studies.

Cu exists widely in nature and can enter the human body through air, soil, water and food. It has been reported that Cu was one of the most abundant particulate matter components, and it was consistently quantified in urine (55). Additionally, Cu remains in the upper part of the soil for a few centimeters, and the majority of Cu in water results from the natural runoff of soil Cu (56). Foods with relatively high Cu content include whole grains, dried fruits, mushrooms, beans, etc. The concentration of Cu in food varies from 0.2 to 44 ppm by wet weight (57). As a relatively short half-life, urinary Cu is mostly regarded as a reliable indicator of recent exposure. Therefore, urinary Cu concentration are typically used to assess Cu exposure, as they can integrate multiple exposure sources, such as air, soil, water and food (22, 23).

Albuminuria is an early indicator of glomerular damage, and it can lead to changes in glomerular filtration rate. Some studies have observed the effect of albuminuria on renal function (58). Increased albuminuria can determine whether immediate therapeutic intervention is required (59). Our study revealed a stable positive association between urinary Cu concentration and UACR. In the GLM, urinary Cu concentration was positively associated with UACR (β = 0.36, 95% CI: 0.25, 0.46) after adjusting for age, sex, BMI, education level, smoking status, drinking status, hypertension and diabetes. The GM (GSD) of the adjusted urinary Cu concentration was 1.08 (1.75) μg/g creatinine, which was lower than other studies (60). A cross-sectional analysis from China (Taiwan, *N* = 2,447 adults) showed that urinary Cu (15 μg/L) concentration was positively associated with albuminuria (36). Consistently, another China Study [Hunan, *N* = 3,553 adults] demonstrated positive dose-response relationships between urinary Cu concentration (median: 14.57 μg/L) and abnormal eGFR (odds ratio [OR] = 3.70, 95% CI: 1.92, 7.14) (19). Johnson et al. (61) found that among participants with CKD, a majority of participants were female. This study supported this finding in females, resulting in a higher likelihood of albuminuria. Urinary Cu concentration were found to be higher in males than in females in a study conducted in Wuhan among 226 individuals with a mean age of 43.6 years (60), which indicated more chronic exposure for males.

Cu is an essential trace element and cofactor of several enzymes, and it is involved in physiological pathways such as heme synthesis and iron absorption (62). Previous histopathological examination found that Cu could cause kidney dysfunction, characterized by degeneration of tubule cells (apoptotic or necrotic) (63, 64). An experimental study showed a time-dependent increase in apoptosis in chickens exposed to Cu. Increased apoptosis index and leakage of blood urea nitrogen (BUN) and creatinine suggest that Cu may lead to kidney dysfunction (65). Previous studies have shown that Cu can induce kidney dysfunction through oxidative damage, mitochondria damage (65, 66). The toxicity of excessive Cu is primarily involved in the generation of reactive oxygen species (ROS), which in turn lead to inhibited levels of antioxidant enzyme and lipid peroxidation, ultimately causing the elevations of BUN and creatinine may lead to the elevations of BUN and creatinine (67, 68). In addition, excessive endogenous ROS alters mitochondrial inner membrane structure, resulting in leakage of cytochrome-c, which further activates caspases (69). Caspase-3 executes the apoptosis phase and acts as a significant contributor to nephrotoxicity (70).

Cu is an essential trace element and cofactor of several enzymes, and it is involved in physiological pathways such as heme synthesis and iron absorption (62). Previous histopathological examination found that Cu could cause kidney dysfunction, characterized by degeneration of tubule cells (apoptotic or necrotic) (63, 64). An experimental study showed a time-dependent increase in apoptosis in chickens exposed to Cu. Increased apoptosis index and leakage of blood urea nitrogen (BUN) and creatinine suggest that Cu may lead to kidney dysfunction (65). Previous studies have shown that Cu can induce kidney dysfunction through oxidative damage, mitochondria damage (65, 66). The toxicity of excessive Cu is primarily involved in the generation of reactive oxygen species (ROS), which in turn lead to inhibited levels of antioxidant enzyme and lipid peroxidation, ultimately causing the elevations of BUN and creatinine may lead to the elevations of BUN and creatinine (67, 68). In addition, excessive endogenous ROS alters mitochondrial inner membrane structure, resulting in leakage of cytochrome-c, which further activates caspases (69). Caspase-3 executes the apoptosis phase and acts as a significant contributor to nephrotoxicity (70).

There are some limitations in our study. The long-term clinical outcomes and causal relationships could not be confirmed given the cross-sectional study design. Long-term cohort studies are needed to verify our results, including serial measurements of heavy metals and renal function. Besides, this study was carried out among elderly over 60 years old and in a single city, so the extrapolation of the results needs to be further discussed.

The present study also has several strengths. First, we focused on an early indicator of glomerular damage, UACR, which is noninvasive and provides an early warning. Second, multiple statistical methods, including GLM, BKMR and qgcomp, were used to verify the stability of the results. Third, the study conducted in areas with high levels of air pollution and during periods of high levels of air pollution provides evidence to further understand the effects of urine metals on humans.

## Conclusion

In the area and time of serious air pollution, we found a stable positive association between urinary Cu and UACR. Future research may focus on prospective studies of mixture exposure and renal function, as well as a better understanding of the complex biological mechanisms of chemical mixtures affecting the kidneys and other organ systems.

## Data Availability Statement

The raw data supporting the conclusions of this article will be made available by the authors, without undue reservation.

## Ethics Statement

The studies involving human participants were reviewed and approved by Institutional Ethics Committees of the Institute of Basic Medicine in the Chinese Academy of Medical Sciences. The patients/participants provided their written informed consent to participate in this study.

## Author Contributions

AL and JZ: conceptualization, investigation, data curation, methodology, data analysis, writing—original draft preparation, reviewing and editing, and funding acquisition. LL, YM, QZ, MZ, JX, and XG: resources, data collection—organization and completion of filed research work, and writing—reviewing and editing. QX: writing—reviewing and editing, supervision, and funding acquisition. All authors contributed to the article and approved the submitted version.

## Funding

This study was supported by the China Medical Board (Grant No. 15-230), China Prospective cohort study of Air pollution and health effects in Typical areas (C-PAT) (Grant No. MEE-EH-20190802), the Fundamental Research Funds for the Central Universities (Grant No. 3332019147), and Peking Union Medical College Graduate Innovation Fund (No. 2019-1004-02). These funders did not participate in the organization of the study design, data collection, analysis, or writing and did not impose any restrictions regarding the publication of the report.

## Conflict of Interest

The authors declare that the research was conducted in the absence of any commercial or financial relationships that could be construed as a potential conflict of interest.

## Publisher's Note

All claims expressed in this article are solely those of the authors and do not necessarily represent those of their affiliated organizations, or those of the publisher, the editors and the reviewers. Any product that may be evaluated in this article, or claim that may be made by its manufacturer, is not guaranteed or endorsed by the publisher.
