# Supplementary material for: Association of Metals and Metalloids With Urinary Albumin/Creatinine Ratio: Evidence From a Cross-Sectional Study Among Elderly in Beijing
Source: Front Public Health. 2022 Mar 31;10:832079. doi: 10.3389/fpubh.2022.832079 (PMC9008350; doi:10.3389/fpubh.2022.832079)
Supplement: Supplementary file 1 [file Data_Sheet_1.pdf]

Supplementary Materials

Supplementary Table 1. The validation parameters for urinary metals and metalloids.

Elements	Sample measurement（μg/L）	Addition standard（μg/L）	Measurement after addition（μg/L）	Recovery rate（%）	Intra-assay coefficients of variation (%)	Inter-assay coefficients of variation (%)
Al	1.278	2.0	3.552	113.7	4.08	3.94
As	1.856	2.0	3.835	98.9	1.35	4.41
Ba	0.535	2.0	2.634	104.9	2.63	4.70
Cd	0.264	2.0	2.1567	94.7	1.63	2.17
Co	0.317	2.0	2.404	104.3	2.78	4.30
Cr	0.043	2.0	1.846	90.2	2.96	6.54
Cs	1.464	2.0	3.467	100.1	3.54	3.65
Cu	1.255	2.0	3.241	99.3	2.01	4.87
Fe	3.073	2.0	5.078	100.2	2.16	6.65
Mn	0.017	2.0	2.198	109.1	2.55	3.08
Ni	0.514	2.0	2.392	93.9	2.12	5.68
Pb	0.320	2.0	2.229	95.4	2.88	4.36
Se	6.016	2.0	8.087	103.5	1.51	4.74
Sr	15.962	10.0	25.911	99.5	1.64	4.00
Zn	116.401	50.0	170.620	102.5	3.44	4.35

Supplementary Table 2. Linearity, range, limit of detection and limit of quantification of urinary elements.

Elements	Range (μg/L)	Regression equation	r	Limit of detection (μg/L)	limit of quantification (μg/L)
Al	0～50	y=0.143x-0.093	0.9999	0.050	0.167
As	0～50	y=0.008 69x+0.000 396	1.0000	0.018	0.060
Ba	0～10	y=0.018x+0.000 094 0	0.9999	0.015	0.050
Cd	0～10	y=0.006x-0.000 154	1.0000	0.003	0.010
Co	0～10	y=0.529x+0.005 7	0.9998	0.013	0.043
Cr	0～10	y=0.353x+0.004 8	1.0000	0.025	0.083
Cs	0～10	y=0.083x+0.000 237	1.0000	0.002	0.007
Cu	0～10	y=0.161x+0.019	0.9993	0.165	0.550
Fe	0～300	y=0.535x-0.075	0.9999	0.045	0.150
Mn	0～10	y=0.569x+0.001 9	0.9999	0.014	0.047
Ni	0～10	y=0.097x+0.003	0.9997	0.028	0.093
Pb	0～10	y=0.120x-0.054	1.0000	0.020	0.067
Se	0～300	y=0.008 68x+0.000 396	0.9999	0.137	0.457
Sr	0～300	y=0.087x-0.053	1.0000	0.064	0.213
Zn	0～500	y=0.100 6x+0.312	0.9995	0.173	0.577

Supplementary Table 3. Association between urinary metals and metalloids concentration and UACR.

Elements	Tertiles of urinary elements (μg/g creatinine) [a]		
	T1	T1	T3
Al	[0.691,3.050]	(3.050,6.480]	(6.480,105.000]
β (95%CI) [b]	ref.	0.01 (−0.20, 0.21)	0.01 (−0.19, 0.22)
As	[0.007,1.640]	(1.640,3.000]	(3.000,32.300]
β (95%CI) [b]	ref.	−0.06 (−0.26, 0.14)	0.12 (−0.10, 0.35)
Ba	[0.004,0.203]	(0.203,0.644]	(0.644,28.300]
β (95%CI) [b]	ref.	0.04 (−0.16, 0.24)	0.15 (−0.05, 0.35)
Cd	[0.001,0.050]	(0.050,0.097]	(0.097,0.481]
β (95%CI) [b]	ref.	−0.05 (−0.26, 0.15)	0.04 (−0.16, 0.24)
Co	[0.012,0.068]	(0.068,0.182]	(0.182,1.930]
β (95%CI) [b]	ref.	0.03 (−0.18, 0.23)	0.13 (−0.10, 0.35)
Cr	[0.086,0.224]	(0.224,0.351]	(0.351,2.260]
β (95%CI) [b]	ref.	−0.05 (−0.25, 0.16)	0.05 (−0.15, 0.26)
Cs	[0.002,0.799]	(0.799,1.240]	(1.240,12.500]
β (95%CI) [b]	ref.	0.02 (−0.19, 0.23)	0.19 (−0.02, 0.40)
Cu	[0.060,0.889]	(0.889,1.440]	(1.440,32.900]
β (95%CI) [b]	ref.	0.14 (−0.04, 0.33)	**0.55 (0.37, 0.73)**
Fe	[0.427,2.800]	(2.800,5.770]	(5.770,149.000]
β (95%CI) [b]	ref.	0.18 (−0.02, 0.38)	**0.29 (0.10, 0.49)**
Mn	[0.003,0.012]	(0.012,0.075]	(0.075,18.000]
β (95%CI) [b]	ref.	0.09 (−0.12, 0.29)	−0.01 (−0.21, 0.20)
Ni	[0.009,0.266]	(0.266,0.659]	(0.659,6.630]
β (95%CI) [b]	ref.	−0.05 (−0.25, 0.15)	0.07 (−0.15, 0.28)
Pb	[0.005,0.070]	(0.070,0.275]	(0.275,13.900]
β (95%CI) [b]	ref.	−0.08 (−0.29, 0.14)	0.13 (−0.10, 0.36)
Se	[0.496,2.370]	(2.370,5.500]	(5.500,25.500]
β (95%CI) [b]	ref.	0.04 (−0.18, 0.26)	0.12 (−0.10, 0.33)
Sr	[0.205,9.150]	(9.150,15.800]	(15.800,125.000]
β (95%CI) [b]	ref.	−0.02 (−0.22, 0.19)	0.03 (−0.17, 0.23)
Zn	[3.260,39.600]	(39.600,63.200]	(63.200,436.000]
β (95%CI) [b]	ref.	−0.04 (−0.25, 0.16)	0.06 (−0.16, 0.27)

[a] Urinary element concentration was presented as urinary creatinine adjusted data.

[b] Metals and metalloids were included in GLM and adjusted for age, sex, BMI, education levels, smoking status, drinking status, hypertension and diabetes.

Abbreviations: T1: first tertile; T2: second tertile; T3: third tertile; 95% CI: 95% confidence interval.

Boldface type indicates effect estimates were statistically significant, P<0.05.

Supplementary Table 4. Stratified analysis: The association between urinary element and UACR by age and gender.

Element	Age						Gender					
	<65 years			≥65 years			Male			Female		
	β (95% CI)	*P* value	*P* FDR	β (95% CI)	*P* value	*P* FDR	β (95% CI)	*P* value	*P* FDR	β (95% CI)	*P* value	*P* FDR
Al	0.07 (-0.06, 0.20)	0.28	0.84	-0.02 (-0.15, 0.10)	0.72	0.96	-0.01 (-0.16, 0.14)	0.91	0.96	0.01 (-0.11, 0.14)	0.84	0.96
As	0.03 (-0.19, 0.25)	0.81	0.96	0.08 (-0.05, 0.22)	0.23	0.84	0.21 (0.03, 0.39)	0.03	0.30	-0.02 (-0.16, 0.11)	0.72	0.96
Ba	0.03 (-0.06, 0.12)	0.51	0.96	0.05 (-0.04, 0.13)	0.28	0.84	0.00 (-0.11, 0.11)	0.97	0.99	0.05 (-0.02, 0.13)	0.18	0.84
Cd	-0.01 (-0.11, 0.09)	0.82	0.96	0.08 (-0.01, 0.17)	0.10	0.55	0.02 (-0.09, 0.13)	0.76	0.96	0.03 (-0.07, 0.13)	0.56	0.96
Co	0.12 (-0.02, 0.25)	0.10	0.55	-0.01 (-0.14, 0.12)	0.88	0.96	0.07 (-0.07, 0.22)	0.30	0.84	0.00 (-0.12, 0.13)	0.94	0.98
Cr	0.03 (-0.19, 0.25)	0.81	0.96	0.00 (-0.17, 0.18)	0.96	0.99	0.13 (-0.09, 0.35)	0.24	0.84	-0.02 (-0.2, 0.16)	0.85	0.96
Cs	0.23 (-0.02, 0.49)	0.08	0.52	0.08 (-0.08, 0.24)	0.33	0.84	0.07 (-0.19, 0.34)	0.59	0.96	0.09 (-0.06, 0.25)	0.23	0.84
**Cu**	**0.47 (0.24, 0.71)**	<**0.01**	<**0.01**	**0.34 (0.22, 0.46)**	<**0.01**	<**0.01**	**0.46 (0.29, 0.63)**	<**0.01**	<**0.01**	**0.31 (0.17, 0.44)**	<**0.01**	<**0.01**
Fe	0.10 (-0.05, 0.25)	0.21	0.84	0.12 (0.00, 0.25)	0.05	0.44	0.15 (0.00, 0.3)	0.05	0.42	0.12 (-0.02, 0.26)	0.10	0.55
Mn	-0.01 (-0.08, 0.06)	0.70	0.96	-0.02 (-0.09, 0.05)	0.58	0.96	-0.03 (-0.12, 0.05)	0.44	0.94	-0.01 (-0.07, 0.06)	0.87	0.96
Ni	-0.04 (-0.17, 0.09)	0.57	0.96	0.04 (-0.06, 0.13)	0.48	0.96	0.01 (-0.12, 0.14)	0.87	0.96	0.06 (-0.04, 0.16)	0.26	0.84
Pb	0.010 (-0.09, 0.10)	0.92	0.97	0.05 (-0.03, 0.13)	0.23	0.84	0.03 (-0.07, 0.13)	0.53	0.96	0.02 (-0.07, 0.1)	0.68	0.96
Se	0.09 (-0.08, 0.27)	0.30	0.84	0.05 (-0.10, 0.20)	0.50	0.96	0.09 (-0.08, 0.26)	0.32	0.84	0.02 (-0.13, 0.17)	0.79	0.96
Sr	0.03 (-0.16, 0.23)	0.72	0.96	0.05 (-0.11, 0.21)	0.54	0.96	0.05 (-0.17, 0.26)	0.67	0.96	0.04 (-0.11, 0.2)	0.61	0.96
Zn	0.08 (-0.12, 0.29)	0.43	0.94	0.19 (0.01, 0.38)	0.04	0.42	0.18 (-0.06, 0.43)	0.14	0.76	0.16 (-0.01, 0.33)	0.06	0.46

Boldface type indicates significant effect estimates after the P-value corrected by FDR.

Supplementary Table 5. Stratified analysis: The association between urinary element and UACR by smoking and drinking status.

Element	Smoking status						Drinking status					
	Non-smoking			Smoking			Non-drinking			Drinking		
	β (95% CI)	*P* value	P_{FDR}	β (95% CI)	*P* value	P_{FDR}	β (95% CI)	*P* value	P_{FDR}	β (95% CI)	*P* value	P_{FDR}
Al	0.01 (-0.10, 0.12)	0.88	0.96	-0.03 (-0.20, 0.14)	0.74	0.96	0.01 (-0.10, 0.13)	0.83	0.96	-0.04 (-0.20, 0.12)	0.61	0.96
As	0.01 (-0.11, 0.14)	0.86	0.96	0.16 (-0.06, 0.38)	0.15	0.80	0.02 (-0.11, 0.14)	0.77	0.96	0.11 (-0.11, 0.32)	0.34	0.84
Ba	0.02 (-0.05, 0.10)	0.57	0.96	0.02 (-0.10, 0.14)	0.74	0.96	0.03 (-0.04, 0.10)	0.34	0.84	0.01 (-0.11, 0.13)	0.90	0.96
Cd	0.00 (-0.09, 0.09)	0.98	0.99	0.02 (-0.11, 0.16)	0.72	0.96	0.05 (-0.05, 0.14)	0.34	0.84	0.00 (-0.11, 0.11)	1.00	1.00
Co	-0.01 (-0.13, 0.10)	0.84	0.96	0.06 (-0.10, 0.23)	0.46	0.96	0.01 (-0.11, 0.13)	0.83	0.96	0.04 (-0.12, 0.19)	0.64	0.96
Cr	-0.01 (-0.17, 0.15)	0.88	0.96	0.14 (-0.13, 0.41)	0.31	0.84	-0.03 (-0.20, 0.14)	0.70	0.96	0.10 (-0.14, 0.33)	0.42	0.94
Cs	0.09 (-0.06, 0.24)	0.25	0.84	0.09 (-0.20, 0.38)	0.55	0.96	0.09 (-0.06, 0.24)	0.25	0.84	0.07 (-0.19, 0.32)	0.60	0.96
**Cu**	**0.31 (0.19, 0.42)**	**<0.01**	**<0.01**	**0.54 (0.28, 0.80)**	**<0.01**	**<0.01**	**0.31 (0.20, 0.43)**	**<0.01**	**<0.01**	**0.47 (0.25, 0.69)**	**<0.01**	**<0.01**
Fe	0.12 (0.00, 0.24)	0.05	0.42	0.09 (-0.09, 0.27)	0.33	0.84	0.12 (-0.01, 0.25)	0.08	0.52	0.10 (-0.05, 0.26)	0.20	0.84
Mn	-0.01 (-0.07, 0.05)	0.77	0.96	-0.06 (-0.16, 0.03)	0.20	0.84	0.02 (-0.05, 0.08)	0.64	0.96	-0.06 (-0.14, 0.03)	0.18	0.84
Ni	0.04 (-0.06, 0.13)	0.44	0.94	0.04 (-0.11, 0.19)	0.60	0.96	0.04 (-0.05, 0.13)	0.41	0.94	0.05 (-0.09, 0.20)	0.47	0.96
Pb	-0.01 (-0.09, 0.07)	0.86	0.96	0.07 (-0.04, 0.19)	0.21	0.84	0.00 (-0.08, 0.08)	0.99	1.00	0.06 (-0.06, 0.17)	0.33	0.84
Se	0.05 (-0.08, 0.19)	0.43	0.94	0.04 (-0.16, 0.24)	0.69	0.96	0.04 (-0.10, 0.19)	0.56	0.96	0.04 (-0.14, 0.23)	0.64	0.96
Sr	0.01 (-0.13, 0.15)	0.88	0.96	0.11 (-0.16, 0.37)	0.44	0.94	0.03 (-0.11, 0.18)	0.67	0.96	0.12 (-0.11, 0.36)	0.32	0.84
Zn	0.18 (0.03, 0.33)	0.02	0.26	0.05 (-0.26, 0.36)	0.75	0.96	0.21 (0.05, 0.37)	0.01	0.16	0.05 (-0.20, 0.29)	0.71	0.96

Boldface type indicates significant effect estimates after the P-value corrected by FDR.

Supplementary Table 6. Model comparison of the three statistical models.

Model comparison	GLM	qgcomp	BKMR
Assumption	(1) Linear association between exposure and outcome; (2) Normally distributed continuous outcome data	(1) Linear association between exposure and outcome; (2) Normally distributed continuous outcome data	Continuous outcomes were normally distributed
Advantages	Results were straightforward and interpretable	(1) Estimated "mixture effects" are useful in studies of exposure to mixtures such as air pollution, dietary and water pollution; (2) directional homogeneity of effect estimates is unnecessary	(1) Explore nonlinear associations and identify interaction effects; (2) relax parametric assumptions, allows high levels flexibility, free of prespecification of interaction or nonlinear effects; (3) tease out the contributions of each exposure or several exposures from the mixture
Limitations	(1) Limited to explore nonlinear interactions and environmental mixtures; (2) May cause collinearity when strong-correlated variables included simultaneously.	(1) Limited to explore nonlinear interactions; (2) If the underlying model is not smooth, the marginal structural model may not adequately capture the dose–response function.	(1) Unstraightforward results and limited to explore co-exposure effects with both high and low-level pollutants;<br>(2) Formal procedures assessing theimportance of interactions are not available.

Supplementary Table 7. Comparison of trace elements with the published data

References	This study		(Lu et al., 2016)	(Tsai et al., 2021)	(Jin et al., 2018)	(Domingo-Relloso et al., 2019)	(Anual et al., 2021)
N	275		53	2,447	3,612	1,171	817
Age (year)	68.9 ± 6.8[a]		18-28[b]	55.1± 13.2 [a]	38.7 (38.1–39.3) [c]	15-85 [b]	≥18 [b]
Region	Beijing, China		Guangzhou, China	Taiwan, China	USA	Spain	Malaysian
Concentration unit	µg/L	µg/g creatine	µg/L	µg/L	µg/L	µg/g creatine	µg/L
Al	38.840 [d]	4.358 [d]	112.2 [e]	-	-	-	-
As	18.979 [d]	2.063 [d]	54.41 [e]	78.9 (45.6-142.0) [c]	10.38 [d]	-	48.21 [d]
Ba	2.848 [d]	0.307 [d]	-	-	1.32 [d]	58.44 (31.71, 103.61) [c]	-
Cd	0.437 [d]	0.049 [d]	1.076 [e]	0.8 (0.3-1.3) [c]	0.33 [d]	0.38 (0.23, 0.64) [c]	0.32 [d]
Co	0.926 [d]	0.104 [d]	0.348 [e]	-	0.35 [d]	0.23 (0.13, 0.48) [c]	-
Cr	2.386 [d]	0.274 [d]	1.732 [e]	0.1 (0.1-0.1) [c]	-	3.58 (2.27, 5.88) [c]	-
Cs	8.171 [d]	0.947 [d]	-	-	4.71 [d]	-	-
Cu	9.589 [d]	1.082 [d]	16.17 [e]	15 (10–20) [c]	-	6.06 (3.78, 9.74) [c]	-
Fe	34.018 [d]	3.903 [d]	44.76 [e]	-	-	-	-
Mn	0.307 [d]	0.031 [d]	2.11 [e]	1.7 (0.9–3.0) [c]	-	-	-
Ni	2.852 [d]	0.311 [d]	8.15 [e]	2.4 (1.5–3.7) [c]	-	-	4.37 [d]
Pb	1.090 [d]	0.118 [d]	5.78 [e]	-	0.61 [d]	-	0.8 [d]
Se	29.228 [d]	3.391 [d]	22 [e]	-	-	-	-
Sr	98.862 [d]	11.499 [d]	218.2 [e]	-	-	-	-
Zn	413.283 [d]	46.847 [d]	-	-	-	183.16 (95.85, 341.73) [c]	

The value of age and concentration of metals was presented inconsistent due to the inconsistent presentation in the original study.

[a] Mean ± standard deviation. [d] Range. [c] median (IQR). [d] Geometric mean. [e] Mean.

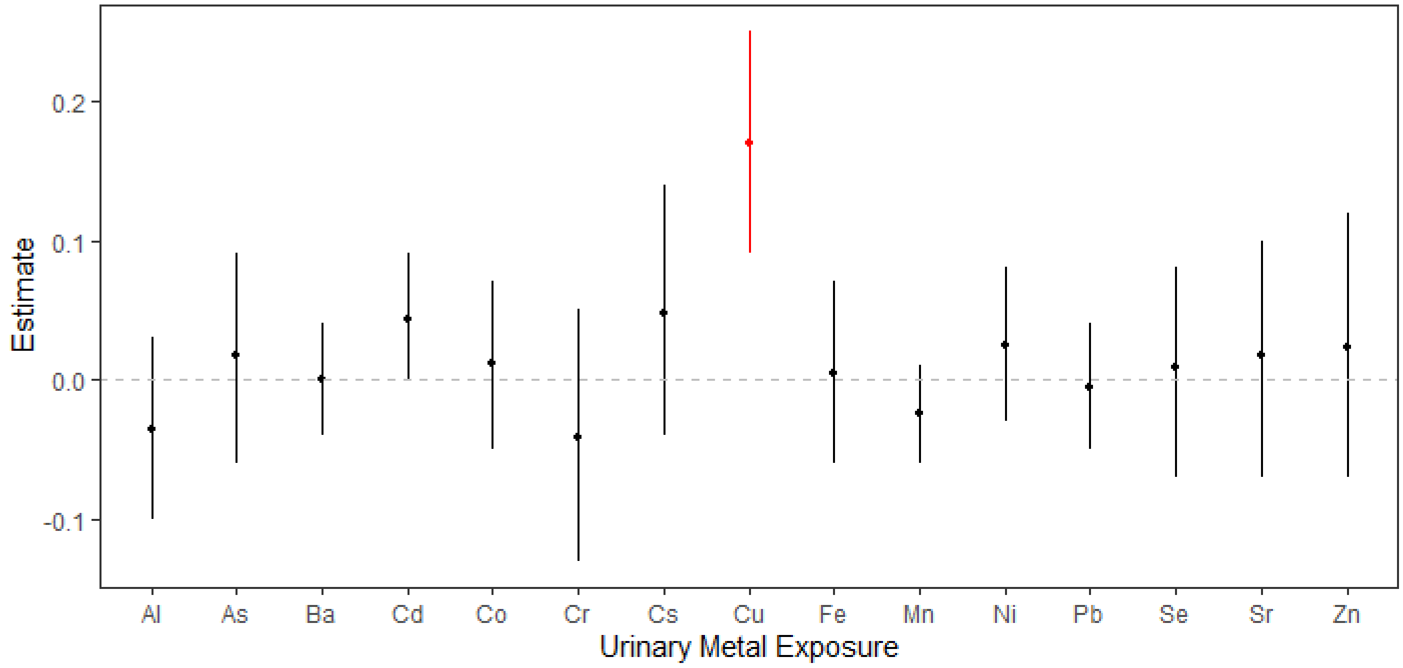

Supplementary Figure 1. Estimated associations between urinary elements and UACR in generalized linear model[a] .
[a]The model was adjusted for age, sex, BMI, education levels, smoking status, drinking status, hypertension, diabetes and CKD status.

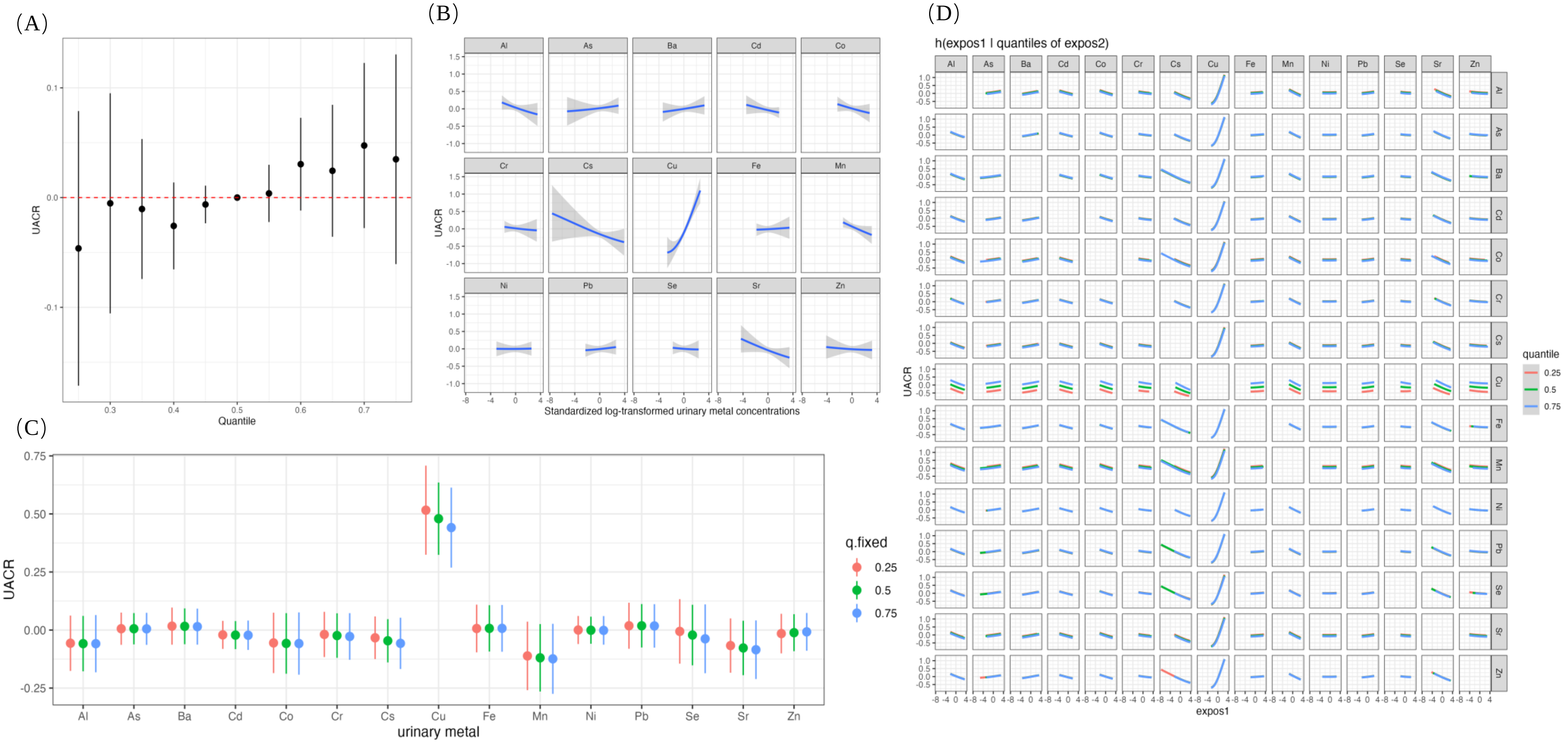

Supplementary Figure 2. Joint effect of the urinary metal and metalloid mixture on UACR by Bayesian Kernel Machine Regression after excluded the outliers of urinary Cu concentration which above the mean concentration ±3 SD [a].

(A) Overall associations between the urinary metal and metalloid and UACR. (B) Univariate exposure-response curves for individual metal. (C) The single-exposure effect of each metal. (D) Bivariate exposure response functions for each of urinary metal and metalloid.

[a] The Model was adjusted for age, sex, BMI, education levels, smoking status, drinking status, hypertension and diabetes.

Reference

Anual, Z. F., N. Mohammad Sham, R. Ambak, F. Othman and R. Shaharudin (2021). "Urinary Concentrations of Metals and Metalloids in Malaysian Adults." Expo Health **13**(3): 391-401.

Domingo-Relloso, A., M. Grau-Perez, L. Briongos-Figuero, J. L. Gomez-Ariza, T. Garcia-Barrera, A. Dueñas-Laita, J. F. Bobb, F. J. Chaves, M. A. Kioumourtzoglou, A. Navas-Acien, J. Redon-Mas, J. C. Martin-Escudero and M. Tellez-Plaza (2019). "The association of urine metals and metal mixtures with cardiovascular incidence in an adult population from Spain: the Hortega Follow-Up Study." Int J Epidemiol **48**(6): 1839-1849.

Jin, R., X. Zhu, M. J. Shrubsole, C. Yu, Z. Xia and Q. Dai (2018). "Associations of renal function with urinary excretion of metals: Evidence from NHANES 2003-2012." Environ Int **121**(Pt 2): 1355-1362.

Lu, S., L. Ren, J. Fang, J. Ji, G. Liu, J. Zhang, H. Zhang, R. Luo, K. Lin and R. Fan (2016). "Trace elements are associated with urinary 8-hydroxy-2'-deoxyguanosine level: a case study of college students in Guangzhou, China." Environ Sci Pollut Res Int **23**(9): 8484-8491.

Tsai, H. J., C. H. Hung, C. W. Wang, H. P. Tu, C. H. Li, C. C. Tsai, W. Y. Lin, S. C. Chen and C. H. Kuo (2021). "Associations among Heavy Metals and Proteinuria and Chronic Kidney Disease." Diagnostics (Basel) **11**(2).
